# Application of the Khorana score for cancer-associated thrombosis prediction in patients of East Asian ethnicity undergoing ambulatory chemotherapy

**DOI:** 10.1186/s12959-023-00505-3

**Published:** 2023-06-05

**Authors:** Hyerim Ha, Yeh-Hee Ko, Kwangsoo Kim, Junshik Hong, Gyeong-Won Lee, Seong Hyun Jeong, Soo-Mee Bang, Sung-Soo Yoon

**Affiliations:** 1 Department of Internal Medicine, Inha University College of Medicine, Inha University Hospital, Incheon, Republic of Korea; 2 Department of Applied Statistics, Yonsei University, Seoul, Republic of Korea; 3 Transdisciplinary Department of Medicine & Advanced Technology, Department of Medicine, College of Medicine, Seoul National University Hospital, Seoul National University, 101 Daehak-ro, Jongno-gu, Adjunct, Seoul, Republic of Korea; 4 Department of Medicine, Seoul National University College of Medicine, Seoul, Republic of Korea; 5 Department of Internal Medicine, Seoul National University Hospital, Seoul National University College of Medicine, 101 Daehak-ro, Jongno-gu, Seoul, Republic of Korea; 6 Cancer Research Institute, Seoul National University College of Medicine, Seoul, Republic of Korea; 7 Division of Hematology-Oncology, Department of Internal Medicine, Gyeongsang National University Hospital, Gyeongsang National University College of Medicine, Jinju, Republic of Korea; 8 Department of Hematology-Oncology, Ajou University School of Medicine, Suwon, Republic of Korea; 9 Department of Internal Medicine, Seoul National University College of Medicine, Seoul National Bundang Hospital, Seongnam, Republic of Korea

**Keywords:** Khorana score, Cancer-associated thrombosis, Venous thrombosis, Asian

## Abstract

**Background:**

The Khorana score (KS) has not been well studied in East Asian cancer patients, who have different genetic backgrounds for inherited thrombophilia, body metabolism, and cancer epidemiology.

**Methods:**

By using the Common Data Model, we retrospectively collected deidentified data from 11,714 consecutive newly diagnosed cancer patients who underwent first-line chemotherapy from December 2015 to December 2021 at a single institution in Korea, and we applied the KS for cancer-associated thrombosis (CAT) prediction. Age at diagnosis, sex, and use of highly thrombogenic chemotherapeutics were additionally investigated as potential risk factors for CAT development.

**Results:**

By 6 months after chemotherapy initiation, 207 patients (1.77%) experienced CAT. Only 0.4% had a body mass index (BMI) ≥ 35 kg/m^2^ and changing the cutoff to 25 kg/m^2^ improved the prediction of CAT. Age ≥ 65 years and the use of highly thrombogenic chemotherapeutics were independently associated with CAT development. KS values of 1 ~ 2 and ≥ 3 accounted for 52.3% and 7.6% of all patients, respectively, and the incidence of CAT in these groups was 2.16% and 4.16%, respectively, suggesting a lower incidence of CAT in the study population than in Westerners. The KS component regarding the site of cancer showed a good association with CAT development but needed some improvement.

**Conclusion:**

The KS was partially validated to predict CAT in Korean cancer patients undergoing modern chemotherapy. Modifying the BMI cutoff, adding other risk variables, and refining the use of cancer-site data for CAT risk prediction may improve the performance of the KS for CAT prediction in East Asian patients.

**Supplementary Information:**

The online version contains supplementary material available at 10.1186/s12959-023-00505-3.

## Background

Patients with cancer have a higher incidence of venous thromboembolism (VTE) than the normal population. Chemotherapy apparently increases the risk of cancer-associated VTE (CAT) by various mechanisms [1]. The development of CAT affects the outcome of anticancer treatment, including interruption or discontinuation of chemotherapy, which may reduce the survival rate of patients [1]. Aside from disease progression, CAT is one of the leading causes of death in patients with cancer [1].

Several studies have been conducted to evaluate the role of medical prophylaxis for VTE in cancer patients undergoing chemotherapy [2–4]. The Phase III SAVE-ONCO trial examined the ultra-low-molecular-weight heparin (ULMWH) semuloparin versus placebo for the prevention of CAT in ambulatory cancer patients undergoing chemotherapy [2]. They reported a reduction in CAT incidence from 3.4% in the placebo arm to 1.2% in the semuloparin arm (P < 0.001). However, semuloparin thromboprophylaxis was not approved for cancer patients undergoing chemotherapy because the degree of efficacy was only modest, the absolute incidence of CAT was low even in the placebo arm, and there remained concerns for bleeding complications. This taught us that thromboprophylaxis should be confined to the portion of patients in whom the risk of CAT is significantly high and exceeds that of bleeding complications.

The Khorana scoring system comprises five parameters: site of cancer; body mass index (BMI); and three prechemotherapy complete blood cell count (CBC) parameters, namely, leukocyte count, platelet count, and hemoglobin level [5, 6]. Expanded scoring models with the addition of several other factors, such as platinum chemotherapeutics (the PROTECHT score) or P-selectin and D-dimer (the Vienna CATS score), followed [7, 8]. The CONKO score, which included performance status instead of BMI, was also suggested. A prospective cohort study [9] compared these scoring systems, but none of them excelled the Khorana score (KS). At present, the KS is the most widely used prediction system for CAT: recent pivotal Phase III trials such as AVERT and CASSINI adopted KS in their inclusion criteria and as a risk stratification factor in randomization [3, 4].

However, most studies on the development and validation of KS have been conducted in Western countries, and this scoring system has scarcely been evaluated or validated in Asian populations. Chen and Khorana analyzed patients who enrolled in the control arm of the SAVE-ONCO trial. Among 1,604 patients in the placebo arm, 277 (17%) were Asian, and there was no difference in CAT risk between Asian and White participants (adjusted hazard ratio: 0.92, 95% confidence interval: 0.43–1.94; *P* = 0.82). However, that study was a post hoc analysis of data from the U.S., where Asians are a small proportion of the population [10]. A recent study tried to validate the KS from a single-center registry of 27,687 Japanese cancer patients over 12 years [11]. It is a well-conducted study that provides important information on the features of CAT in Asians. However, the role of KS in ambulatory Asian cancer patients undergoing chemotherapy still needs to be defined because only 31.9% of the patients received chemotherapy in the Japanese study [11].

Asians have a lower prevalence of VTE than the Western population. This is attributed to a lower prevalence of predisposing genetic factors, such as factor V Leiden, thrombomodulin, and prothrombin gene variants [12]. Considering this fact, it is not clear whether the incidence and characteristics of CAT in Asians are comparable to those in Westerners. In regard to BMI, World Health Organization (WHO) experts adopted adjusted cutoff points for the classification of weight status in Asian, since they usually have lower weights than Westerners [13]. In addition, since the characteristics of each cancer site, including incidence, mortality, and detailed subtypes, vary among geographic regions of the world, [14] the risk of CAT by cancer site may need reassessment in different ethnicities.

The purpose of this study is to investigate the usefulness of the current KS for CAT prediction in Korean cancer patients (*i.e.*, a subset of East Asian cancer patients) who underwent first-line chemotherapy and to explore directions for future improvement of the KS by using the Observational Medical Outcomes Partnership (OMOP) Common Data Model (CDM), which enabled precise capture of the patients’ information, including cancer diagnoses, prechemotherapy laboratory data, and medications.

## Methods

### Data source

A CDM is a standardized form of data structure for the effective utilization of data from medical institutes. In other words, a CDM can unify the terms used in each hospital into standard terminology, enabling larger-scale collaborative analyses of observational data. The OMOP-CDM from Observational Health Data Science and Informatics (OHDSI) is now one of the most widely used CDMs [15]. Each institution participating in OHDSI can convert its data into the Standardized Vocabularies for OMOP-CDM, enabling observational studies without the need to share sensitive patient-level data of their own or with other institutions [15].

### Selection of cancer patients

Using the OMOP-CDM, we collected data from cancer patients who underwent chemotherapy from December 1, 2015, to December 31, 2021, at Seoul National University Hospital (SNUH), Seoul, South Korea. Patients were included if they (1) had a diagnosis code of cancer as a main diagnosis, (2) had initiated ambulatory chemotherapy with either the outpatient clinic or a brief admission to the oncology ward within 3 months from the first recording of the cancer diagnosis, (3) were aged 18 years or older, and (4) had data enabling the KS to be calculated. Diagnosis codes for cancer were given according to the Korean Classification of Diseases version 7 (KCD-7), which is a very slightly modified Korean version of the International Classification of Diseases version 10 (ICD-10; Supplementary Table 1). Patients with central nervous system tumors or multiple myeloma were excluded from the analysis, in line with a previous study [5]. Execution of chemotherapy was detected based on the presence of prescription codes of chemotherapeutic agents plus the institutional procedural terminology codes for patient education for first-line ambulatory chemotherapy initiation. Patients who had two or more cancer diagnosis codes; had already been prescribed anticoagulants prior to cancer diagnosis for other reasons (for example, prevention of stroke in patients with atrial fibrillation); or were missing any CBC, height, or weight data were excluded. The flows of patient selection and exclusion are summarized in Supplementary Fig. 1.

### Acquisition of data

The three prechemotherapy CBC variables as well as the body weight and height of the patients as close as possible to the day of chemotherapy initiation were obtained. BMI was calculated as weight in kilograms divided by height in meters squared. The platinum compounds gemcitabine and asparaginase were classified as posing a high risk of CAT development (Supplementary Table 2 A). Patients with diabetes mellitus and hypertension were searched using diagnosis codes (Supplementary Table 3 A and B) and medication codes (Supplementary Table 2B and C). Chronic kidney disease (CKD) is defined as estimated glomerular filtration rate by Modification of Diet in Renal Disease ≤ 60 mL/min/1.73 m² at the time of chemotherapy initiation. Patients who had already been receiving antiplatelet drugs (such as aspirin and clopidogrel; Supplementary Table 2D) for any reasons other than venous thrombosis at the time of chemotherapy initiation were also identified.

### Definition of cancer-associated thrombosis patients

Among the included patients, episodes of newly diagnosed CAT were identified. A patient was counted as having CAT only if he or she had both a newly recorded diagnosis code of VTE (either pulmonary thromboembolism or deep vein thrombosis; Supplementary Table 3 C) plus a newly recorded anticoagulant medication code (Supplementary Table 2E) within 1 week after the emergence of the VTE diagnosis code. CAT risk assessment was based on the incidence of CAT at six months after chemotherapy.

### Statistical analysis

Continuous variables are expressed as the median and standard error. Pearson’s chi-square test or Fisher’s exact test was used for categorical variables, as appropriate. Multivariable logistic regression was used to verify the risk factors for VTE. All statistical analyses were performed using R software, version 3.6.2.

## Results

### Patient characteristics

A total of 11,714 patients with cancer were eligible for the analysis. The median age was 59 years, and 33.5% of patients were ≥ 65 years of age. Most patients had a BMI of < 25 kg/m^2^ (8,619 patients; 73.6%). In contrast, only 42 patients (0.4%) had a BMI of 35 > kg/m^2^, demonstrating a remarkable difference in the BMI distribution of East Asians compared to that of Westerners. Detailed baseline characteristics are summarized in Table 1.

**Table 1:** Baseline characteristics

Characteristics	No. (%)
All patients	11,714 (100)
Age (years)	
Median [SE]	59 [0.1]
< 65	7,784 (66.5)
≥ 65	3,930 (33.5)
Sex	
Female	6,965 (59.5)
Male	4,749 (40.5)
Diabetes mellitus	
Yes	2,364 (20.2)
No	9,350 (79.8)
Hypertension	
Yes	4,493 (38.4)
No	10,521 (61.6)
Chronic kidney disease	
Yes	697 (6.0)
No	11,017 (94.0)
Preexisting antiplatelet agent	
Yes	1,267 (10.8)
No	10,447 (89.2)
Body mass index (kg/m^2^)	
Median [SE]	22.9 [0.03]
< 25.0	8,619 (73.6)
25-29.9	2,690 (23.0)
30-34.9	363 (3.1)
≥ 35.0	42 (0.4)
Platelet count (×10^3^/µL)	
Median [SE]	263 [0.6]
< 350	9,410 (80.3)
≥ 350	2,304 (19.7)
Hemoglobin (g/dL)	
Median [SE]	12.4 [0.6]
< 10	1,151 (9.8)
≥ 10	10,563 (90.2)
Leukocyte count (×10^3^/µL)	
Median [SE]	6.7 [0.1]
< 11	10,675 (91.1)
≥ 11	1,039 (8.9)
Use of high-CAT-risk chemotherapeutics	
Used	6,716 (57.3%)
Not used	4,998 (42.7%)

Abbreviations: SE, standard error; CAT, cancer-associated thrombosis

### Incidence of CAT

Among all patients, the 6-month incidence of CAT was 1.77% (207 out of 11,714 patients). Pancreatic, biliary, and kidney cancer showed a ≥ 4% incidence of CAT. The incidence of CAT was 3.10% in patients with stomach cancer, which is defined as a very high CAT risk cancer according to the KS. Lung cancer had a relatively low incidence of CAT (1.89%) at 6 months after ambulatory chemotherapy initiation. Breast cancer, which accounted for the largest proportion of patients, showed a very low incidence of CAT (0.21% by 6 months; Table 2).

**Table 2:** Site of primary cancer and incidence of venous thrombosis by primary cancer site

Site	N (%)	Cumulative number of CAT cases at 3 months	Cumulative numbers of CAT cases at 6 months (%)^a)^	Cumulative numbers of CAT cases at 12 months
H&N cancers^b)^	257 (2.2)	3	4 (1.56%)	5
Esophageal	137 (1.2)	3	5 (3.65%)	5
Stomach	946 (8.1)	22	29 (3.10%)	30
Breast	3,352 (28.6)	2	7 (0.21%)	9
Lung	2,166 (18.5)	31	41 (1.89%)	56
Liver	167 (1.4)	5	5 (2.99%)	8
Biliary	196 (1.7)	7	11 (5.61%)	13
Pancreas	481 (4.1)	15	20 (4.16%)	28
Colorectal	2,236 (19.1)	23	32 (1.43%)	39
Kidney	90 (0.8)	5	6 (6.67%)	6
Bladder	109 (0.9)	1	4 (3.67%)	4
Gynecologic	963 (8.2)	26	33 (3.43%)	41
Prostate	37 (3.2)	1	1 (2.70%)	3
Testis	25 (0.2)	0	0 (0%)	0
Lymphoma	552 (4.7)	6	9 (1.63%)	14
Total	11,714 (100)	150	207 (1.77%)	261

Abbreviations: H&N, head and neck; CAT, cancer-associated thrombosis

(a) among all patients with cancer at the corresponding site; (b) including lip, oral cavity, pharyngeal, laryngeal, and thyroid cancer

### Clinical and laboratory parameters associated with CAT development

CAT development at six months was associated with every parameter defined in the original KS except BMI > 35 kg/m^2^ (Table 3). In addition, patients who were ≥ 65 years of age had a significantly higher incidence of CAT than those < 65 years of age. Sex was not associated with CAT incidence. A change in the BMI cutoff value to ≥ 25 kg/m^2^ showed a good association. In a multivariate logistic regression analysis (Table 4), age ≥ 65 years, BMI ≥ 25 kg/m^2^, hemoglobin < 10 g/dL, leukocytes ≥ 11 × 10^3^/µL, use of high-CAT-risk chemotherapeutics, and high-risk or very high-risk cancer sites were independently associated with the development of CAT. Platelets ≥ 350 × 10^3^/µL did not show an association. Diabetes, hypertension, CKD, and preexisting use of antiplatelet agents was associated with the increased incidence of CAT at 6 months individually (Table 3). However, none of them was an independent risk factor for CAT development at 6 months by multiple regression analysis (Table 4).

**Table 3:** Incidence of cancer-associated thrombosis by clinical variables

	Characteristics	No. of cases with CAT (%)	No. of cases without CAT	P value
Age (years)	< 65	106 (1.4)	7,678	< 0.001
	≥ 65	101 (2.6)	3,829	
Sex	Female	115 (1.7)	6,850	0.248
	Male	92 (2.0)	4,657	
BMI (kg/m^2^)	< 25.0	140 (1.7)	8,479	0.050
	≥ 25.0	67 (2.2)	3,028	
BMI (kg/m^2^)	< 30.0	202 (1.8)	11,107	0.536
	≥ 30.0	5 (1.3)	400	
BMI (kg/m^2^)	< 35.0	206 (1.8)	11,466	0.528^a)^
	≥ 35.0	1 (2.4)	41	
Platelet count (×10^3^/µL)	< 350	149 (1.6)	9,261	0.002
	≥ 350	58 (2.6)	2,246	
Hemoglobin (g/dL)	< 10	34 (3.0)	1,117	0.001
	≥ 10	173 (1.7)	10,390	
Leukocyte count (×10^3^/µL)	< 11	166 (1.6)	10,509	< 0.001
	≥ 11	41 (4.1)	998	
Chemotherapeutics	High CAT risk	153 (2.3)	6,563	< 0.001
	No high CAT risk	54 (1.1)	4,944	
Site of cancer^b)^	Low CAT risk	71 (1.1)	6,401	< 0.001
	High CAT risk	87 (2.3)	3,728	
	Very high CAT risk	49 (3.6)	1,378	
Diabetes mellitus	Yes	56 (2.4)	2,308	0.013
	No	151 (1.6)	9,199	
Hypertension	Yes	105 (2.3)	4,388	< 0.001
	No	102 (1.0)	10,419	
Chronic kidney disease	Yes	23 (3.3)	674	0.002
	No	184 (1.7)	10,833	
Preexisting antiplatelet use	Already used	38 (22)	1,229	< 0.001
	Not used	169 (1.6)	10,278	

Abbreviations: CAT, cancer-associated thrombosis; BMI, body mass index;

(a) Fisher’s exact test; (b) according to the Khorana score

**Table 4:** Multiple regression analysis for the risk of developing cancer-associated venous thrombosis

Characteristics	Beta (SE)	OR (95% CI)	P value
(Constant)	-5.19 (0.18)	0.006 (0.004–0.008)	< 0.001
Age ≥ 65 vs. < 65 years	0.47 (0.14)	1.60 (1.21–2.12)	0.001
BMI ≥ 25 vs. < 25 kg/m^2^	0.43 (0.15)	1.53 (1.13–2.06)	0.005
Platelet count ≥ 350 vs. < 350 × 10^3^/µL	0.24 (0.16)	1.28 (0.92–1.75)	0.137
Hemoglobin < 10 vs. ≥ 10 g/dL	0.40 (0.20)	1.49 (0.99–2.15)	0.043
Leukocyte count ≥ 11 vs. < 11 × 10^3^/µL	0.72 (0.19)	2.06 (1.41–2.93)	< 0.001
CAT risk according to chemotherapeutics High risk vs. not high risk	0.46 (0.17)	1.59 (1.15–2.22)	0.006
CAT risk according to cancer site			
- High vs. low risk	0.46 (0.17)	1.58 (1.14–2.20)	0.006
- Very high vs. low risk	0.97 (0.20)	2.64 (1.79–3.87)	< 0.001
Diabetes mellitus yes vs. no	-0.08 (0.17)	0.92 (0.65–1.28)	0.629
Hypertension yes vs. no	0.18 (0.16)	1.19 (0.82–1.62)	0.264
Chronic kidney disease yes vs. no	0.26 (0.24)	1.30 (0.80–2.02)	0.270
Preexisting antiplatelet yes vs. no	0.18 (0.20)	1.20 (0.80–1.75)	0.369

Abbreviations: OR, odds ratio; CI, confidence interval; BMI, body mass index; CAT, cancer-associated thrombosis

### Application of the Khorana score in korean cancer patients

When the KS was applied, 6,023 out of 11,714 patients (52.3%) were found to have an intermediate CAT risk (KS score of 1 or 2), and their 6-month incidence of CAT was 2.26%. In addition, 890 patients (7.6%) were stratified into the high-risk group (score ≥ 3), and their 6-month incidence of CAT was 4.16% (Table 5).

**Table 5:** Cancer-associated venous thrombosis risk by Khorana score for the analyzed patients

Khorana score (KS)	Number of patients (%)	Patients without VTE	Patients with VTE (% in each score group)
0 (low risk)	4,801 (41.0%)	4,764	37 (0.77%)
1 ~ 2 (intermediate risk)	6,023 (52.3%)	5,890	133 (2.26%)
1	3,699 (31.6%)	3,624	75 (2.03%)
2	2,324 (19.8%)	2,266	58 (2.50%)
≥ 2^a)^	3,214 (27.4%)	3,119	95 (2.96%)
≥ 3 (high risk)	890 (7.6%)	853	37 (4.16%)
Total	11,714 (100%)	11,507	207 (1.79%)

a) A Khorana score ≥ 2 was an inclusion criterion in previous Phase III trials such as AVERT and CASSINI

## Discussion

In this study, we investigated the incidence of and risk factors for CAT in newly diagnosed Korean cancer patients who underwent first-line modern (after December 2015) chemotherapy; the KS was partially validated to predict CAT, but we also found room for improvement in its application to East Asians.

BMI is a well-known predictive indicator of VTE,[16] and patients with BMI ≥ 35 kg/m^2^ have a 1-point increase in their risk score under the KS scoring system. However, BMI shows significantly different distribution patterns between the Western and Eastern populations [13]. In Western countries, a BMI ≥ 30 kg/m^2^ is considered obese, and this category accounts for 20 ~ 30% of the population. In contrast, only 2 ~ 4% of the population has a BMI ≥ 30 kg/m^2^ in East Asia, including Korea and Japan [17]. Another study compared the BMI of 274 cases in Western countries and 437 cases in Eastern countries: the median BMI was 26.3 kg/m^2^ in the West but 22.5 kg/m^2^ in the East [18]. In our analysis, patients with a BMI > 35 kg/m^2^ accounted for only 0.4%, and lowering the cutoff to 25 kg/m^2^ improved the prediction of CAT incidence in the first 6 months after chemotherapy. This appears to be due to the exceedingly small number of cases of BMI ≥ 35 kg/m^2^, rather than a true lack of association between very high BMI and CAT development.

The three CBC variables were included in the original KS system because they showed an independent association with CAT development by multivariate analyses in Khorana’s early studies [5, 6]. In our study, a platelet count ≥ 350 × 10^3^/µL was not independently associated with CAT development at 6 months. Considering that there have been basic and clinical data explaining the association between thrombocytosis and VTE development,[1] additional studies investigating the degree of association between thrombocytosis and CAT development in East Asians are needed.

Age is known as an independent risk factor for VTE in a variety of circumstances, including major surgery, atrial fibrillation, and polycythemia vera [19, 20]. In retrospective studies for the development of Khorana’s prediction model, age ≥ 65 years was not a significant predictive factor for CAT development in univariate and multivariate analyses, and was thus excluded from the Khorana scoring system [5, 6]. However, several studies have still shown this association [21]. In cancer patients with neutropenia, age ≥ 65 years was the clinical factor most frequently associated with CAT, and another study found that the risk of VTE approximately doubled in elderly cancer patients aged 70 years or older [22, 23]. In our study, age ≥ 65 years was significantly associated with CAT development. One possible hypothesis is that because Asians have a substantially lower incidence of inherited thrombophilia,[24] they may have a relatively high proportion of later-onset venous thrombosis attributable to acquired VTE risk factors as they age.

In a meta-analysis [25] of 45 articles and eight abstracts published between 2008 and 2018 (N = 27,849), 17% of ambulatory cancer patients were classified in the high-risk group according to the KS. Of these patients, 11.5% (95% CI, 8.8–13.8) developed CAT at the 6-month follow-up. Another meta-analysis of individual patient data (N = 3,293) found that 23% of patients were high-risk patients, and the 6-month incidence of CAT among them was 9.8%. [26] However, in our analysis, only 7.6% of evaluable patients had a KS ≥ 3, and among them, only 4.16% exhibited CAT within 6 months. Considering that the proportions of cancers occurring at different sites of cancer did not greatly differ between previous Western studies and our study, the evidence strongly suggests that the incidence of CAT, at least in the setting of initial chemotherapy in ambulatory cancer patients, is substantially lower in people of East Asian ethnicity than in the Western population. The AVERT and CASSINI Phase III trials used a KS ≥ 2 instead of ≥ 3 as an inclusion criterion, and the 180-day incidence rates of CAT in the placebo groups of these two trials were 10.2% and 8.8%, respectively. In contrast, the 6-month incidence of CAT at 6 months among patients with a KS ≥ 2 was only 2.96% in our study, also suggesting a lower incidence.

Patients with gastric and pancreatic cancers were classified into the ‘very high’ risk group according to the KS because their risk of CAT development was at least 3 times the average risk for the analyzed population [5]. In the current study, the incidence of VTE at 6 months was considerably high in pancreatic cancer (15 out of 481 patients: 4.16%). Intriguingly, patients with stomach cancer showed a lower 6-month incidence of CAT (29 out of 946 patients: 3.10%). This could be explained by several postulations. First, the characteristics of stomach cancer in East Asians are different from those in Western countries [27]. In Western countries, the majority of gastric cancers occur at the esophagogastric junction area, whereas in East Asia, the antrum and pylorus are the most common regions. In addition, the age at the time of stomach cancer diagnosis is younger in Asian patients than in Western patients [28]. Thus, although the diseases are placed in the same category, ‘stomach cancer,’ their biology may differ. The second potential reason is the higher early detection rate of stomach cancer due to the greater accessibility of endoscopic examination in several East Asian countries, such as Japan and South Korea: [29] the lower tumor burden may have contributed to the lower incidence of CAT [30]. The incidence of CAT in biliary tract cancer (5.61%), which is not noted as ‘high risk’ under the KS, was very high in our patients. On the other hand, the incidence rates of CAT in patients with lymphoma and lung cancer, considered ‘high-risk’ cancers under the KS, were relatively low. This result is in line with a recent meta-analysis reporting that the incidence of CAT was lower than expected in patients with lung cancers [25]. Since we evaluated more recently treated cancer patients, a significant proportion of patients with non-small cell lung cancer would receive tyrosine kinase inhibitors as first-line treatment instead of platinum-containing chemotherapy. Contrary to these discrepancies, the high risk of CAT in gynecologic and bladder cancers and low risk of CAT in breast and colon cancers were reproduced clearly in our work.

Although diabetes and hypertension are suggested as risk factors of VTE [31, 32], the association of CAT and diabetes or hypertension has not been thoroughly studied. A previous study reported the association between CKD and general VTE [33]. However, reduced renal function was not an independent risk factor of CAT in cancer patients enrolled in the Vienna Cancer and Thrombosis cohort study (CATS) [34]. In our analysis, we found that none of them was an independent risk factor for CAT development, suggesting the overwhelming effect of chemotherapy on the development of early phase (within 6 months) CAT in ambulatory cancer patients. The chronic effect of these underlying diseases in cancer patients should be evaluated separately in further studies with long-term follow up.

We are aware of the limitations of our study. First, it is a retrospective study, and some asymptomatic cases of CAT may not be detected without active screening. However, all patients received first-line systemic chemotherapy, and thus, very regular outpatient visits and relatively frequent imaging studies for disease status monitoring or response evaluation were conducted in most of the patients. Second, the original KS combined low hemoglobin with the use of erythropoietin stimulating agents (ESAs). However, we did not include the use of ESA because it is seldom used for the correction of anemia in Korean patients with solid cancers. Third, a small number of patients may have died suddenly due to massive pulmonary embolism, and those cases may not have been detected.

## Conclusion

The Khorana scoring system was partially effective in Korean cancer patients who underwent first-line chemotherapy. The incidence of CAT was generally lower in East Asian cancer patients than in Western patients. A reduced BMI cutoff is required for the East Asian population. Adding new variables, such as age ≥ 65 years and use of high-CAT-risk chemotherapeutics, may improve prediction performance. Although site-specific CAT risk scoring in the original KS was effective in East Asian cancer patients, the severity of CAT risk for each site of cancer may need further investigation. Future prospective studies to establish an optimal CAT prediction tool in East Asian cancer patients are urgently warranted.

## Electronic supplementary material

Below is the link to the electronic supplementary material.

Supplementary Material 1

## Data Availability

The dataset used and/or analyzed during the current study is available from the corresponding authors on reasonable request.
