# Supplementary material for: Application of the Khorana score for cancer-associated thrombosis prediction in patients of East Asian ethnicity undergoing ambulatory chemotherapy
Source: Thromb J. 2023 Jun 5;21:63. doi: 10.1186/s12959-023-00505-3 (PMC10240689; doi:10.1186/s12959-023-00505-3)
Supplement: Supplementary file 1 — Supplementary Material 1 [file 12959_2023_505_MOESM1_ESM.docx]

**Supplementary Table 1. Cancer diagnosis code according to the Korean Standard Classification of Disease version 7**

| Diagnostic code | Disease |
| --- | --- |
| C00-14 | Malignant neoplasm of the lip, oral cavity, and pharynx |
| C15 | Malignant neoplasm of the esophagus |
| C16 | Malignant neoplasm of the stomach |
| C18-20 | Malignant neoplasm of the colon, rectosigmoid junction and rectum |
| C22 | Malignant neoplasm of the liver and intrahepatic bile ducts |
| C23-24 | Malignant neoplasm of the gallbladder, other and unspecified parts of the biliary tract |
| C25 | Malignant neoplasm of the pancreas |
| C32 | Malignant neoplasm of the larynx |
| C33-34 | Malignant neoplasm of the trachea, bronchus, and lung |
| C50 | Malignant neoplasm of the breast |
| C53-56 | Malignant neoplasm of the cervix uteri, corpus uteri, uterus, and ovary |
| C61 | Malignant neoplasm of the prostate |
| C62 | Malignant neoplasm of the testis |
| C64 | Malignant neoplasm of the kidney, except the renal pelvis |
| C67 | Malignant neoplasm of the bladder |
| C73 | Malignant neoplasm of the thyroid gland |
| C81-86 | Hodgkin, follicular, nonfollicular, mature T/NK cell, other and unspecified type of non-Hodgkin, and other specified types of T/NK-cell lymphoma |
| C96 | Other and unspecified malignant neoplasm of lymphoid, hematopoietic, and related tissue |

**Supplementary Table 2. Medication code according to the Anatomical Therapeutic Chemical classification system; (A) Diabetes, (B) Hypertension, (C) Antiplatelet, (D) Anticoagulant, and (E) Anticancer drug**

1. **Anticancer drugs of high venous thrombosis risk**

| Anatomical Therapeutic Chemical code | Medication |
| --- | --- |
| L01XX02 | Asparaginase |
| L01XA02 | Carboplatin |
| L01XA01 | Cisplatin |
| L01BC05 | Gemcitabine |
| L01XA03 | Oxaliplatin |

1. **Diabetes**

| Anatomical Therapeutic Chemical code | Medication |
| --- | --- |
| A10 | Drugs used in diabetes |

1. **Hypertension**

| Anatomical Therapeutic Chemical code | Medication |
| --- | --- |
| C02 | Antihypertensive |
| C08 | Calcium channel blockers |
| C09 | Agents acting on the renin-angiotensin system |

1. **Antiplatelet**

| Anatomical Therapeutic Chemical code | Medication |
| --- | --- |
| B01AC06 | Aspirin |
| B01AC04 | clopidogrel |

1. **Anticoagulant**

| Anatomical Therapeutic Chemical code | Medication |
| --- | --- |
| B01AA03 | Warfarin |
| B01AB01 | Heparin |
| B01AB04 | Dalteparin |
| B01AB05 | Enoxaparin |
| B01AE07 | Dabigatran |
| B01AF01 | Rivaroxaban |
| B01AF02 | Apixaban |
| B01AF03 | Edoxaban |

**Supplementary Table 3. Diagnosis code according to the Korean Standard Classification of Disease version 7; (A) Diabetes, (B) Hypertension and (C)Venous thrombosis**

1. **Diagnosis code of diabetes**

| Diagnosis code | Disease |
| --- | --- |
| E10 | Type 1 diabetes mellitus |
| E11 | Type 2 diabetes mellitus |

1. **Diagnosis code of Hypertension**

| Diagnosis code | Disease |
| --- | --- |
| I10 | Essential hypertension |
| I11 | Hypertensive heart disease |
| I12 | Hypertensive renal disease |
| I13 | Hypertensive heart and renal disease |
| I15 | Secondary hypertension |

1. **Diagnosis code of Venous thrombosis**

| Diagnosis code | Disease |
| --- | --- |
| I26.0 | Pulmonary embolism with mention of acute cor pulmonale  Acute cor pulmonale NOS |
| I26.9 | Pulmonary embolism without mention of acute cor pulmonale  Pulmonary embolism NOS |
| I80.2 | Phlebitis and thrombophlebitis of superficial vessels of lower extremities  Deep vein thrombosis NOS |
| I80.3 | Phlebitis and thrombophlebitis of lower extremities, unspecified  Embolism or thrombosis of lower extremity NOS |
| I82.8 | Phlebitis and thrombophlebitis of other sites |
| I82.9 | Phlebitis and thrombophlebitis of unspecified site |

**Supplementary Figure 1. Patient selection flow**
